# Supplementary material for: Anti-Inflammatory Cytokine Profiles in Thrombotic Thrombocytopenic Purpura—Differences Compared to COVID-19
Source: Int J Mol Sci. 2024 Sep 17;25(18):10007. doi: 10.3390/ijms251810007 (PMC11432022; doi:10.3390/ijms251810007)
Supplement: Supplementary file 1 [file ijms-25-10007-s001.zip › ijms-3181414-supplementary.pdf]

Anti-inflammatory cytokine profiles in thrombotic thrombocytopenic purpura – differences compared to COVID-19

Flóra Demeter[1], György Bihari[1], Dorina Vadicsku[1], György Sinkovits[1], Erika Kajdácsi[1,2], Laura Horváth[1], Marienn Réti[3], Veronika Müller[4], Zsolt Iványi[5], János Gál[5], László Gopcsa[3], Péter Reményi[3], Beáta Szathmáry[6], Botond Lakatos[6], János Szlávik[6], Ilona Bobek[7], Zita Z Prohászka[1], Zsolt Förhécz[1], Tamás Masszi[1], István Vályi-Nagy[3], Zoltán Prohászka[1,2], László Cervenak[1]

[1] Department of Internal Medicine and Haematology, Semmelweis University, Budapest, Hungary
[2] Research Group for Immunology and Haematology, Semmelweis University— HUN-REN-SU (Office for Supported Research Groups), Budapest, Hungary
[3] Department of Haematology and Stem Cell Transplantation, Central Hospital of Southern Pest National Institute of Haematology and Infectious Diseases, Budapest, Hungary
[4] Department of Pulmonology, Semmelweis University, Budapest, Hungary
[5] Department of Anaesthesiology and Intensive Therapy, Semmelweis University, Budapest, Hungary
[6] Department of Infectology, Central Hospital of Southern Pest - National Institute of Haematology and Infectious Diseases, Budapest, Hungary
[7] Department of Anaesthesiology and Intensive Therapy, Central Hospital of Southern Pest - National Institute of Haematology and Infectious Diseases, Budapest, Hungary

SUPPLEMENTARY MATERIAL

METHODS

1. MCP-1 ELISA

For ELISA validation, we used the Human CCL2/MCP-1 DuoSet ELISA kit from R&D Systems, following the manufacturer's instructions. Plasma samples were diluted twofold, and detection was performed using a TECAN Infinite M1000 Pro multifunctional monochromator-based microplate reader.

RESULTS

1. Correlation of MCP-1 concentrations measured by Legendplex and ELISA

To verify our measurements with the Legendplex array, we repeated the measurement of a selected cytokine, MCP-1, with sandwich ELISA. The results from the Legendplex array and ELISA showed a good correlation, with a Spearman r value of 0.5813 (**Figure S2**).

2. Correlation of sCD25 concentrations measured using the Human Inflammation Panel and the Human Covid-19 Cytokine Storm Panel 2 Legendplex kits

Both the Human Inflammation Panel and the Human Covid-19 Cytokine Storm Panel 2 Legendplex kits included sCD25, therefore we measured its concentration twice. To ensure the accuracy of the Legendplex method, we compared the results of these two measurements and found a notable correlation, with a Spearman r value of 0.7063 (**Figure S3**).

3. Correlation of SM concentrations of the Covid 3 and Covid 4 groups

For certain analyses, the Covid 3 and Covid 4 groups were combined (Covid 3-4) and referred to as severe Covid. To validate this approach, we compared SM concentrations of these two severity groups and found a strong correlation, with a Spearman r value of 0.9319 (**Figure S4**).

SUPPLEMENTARY FIGURES

Figure S1.

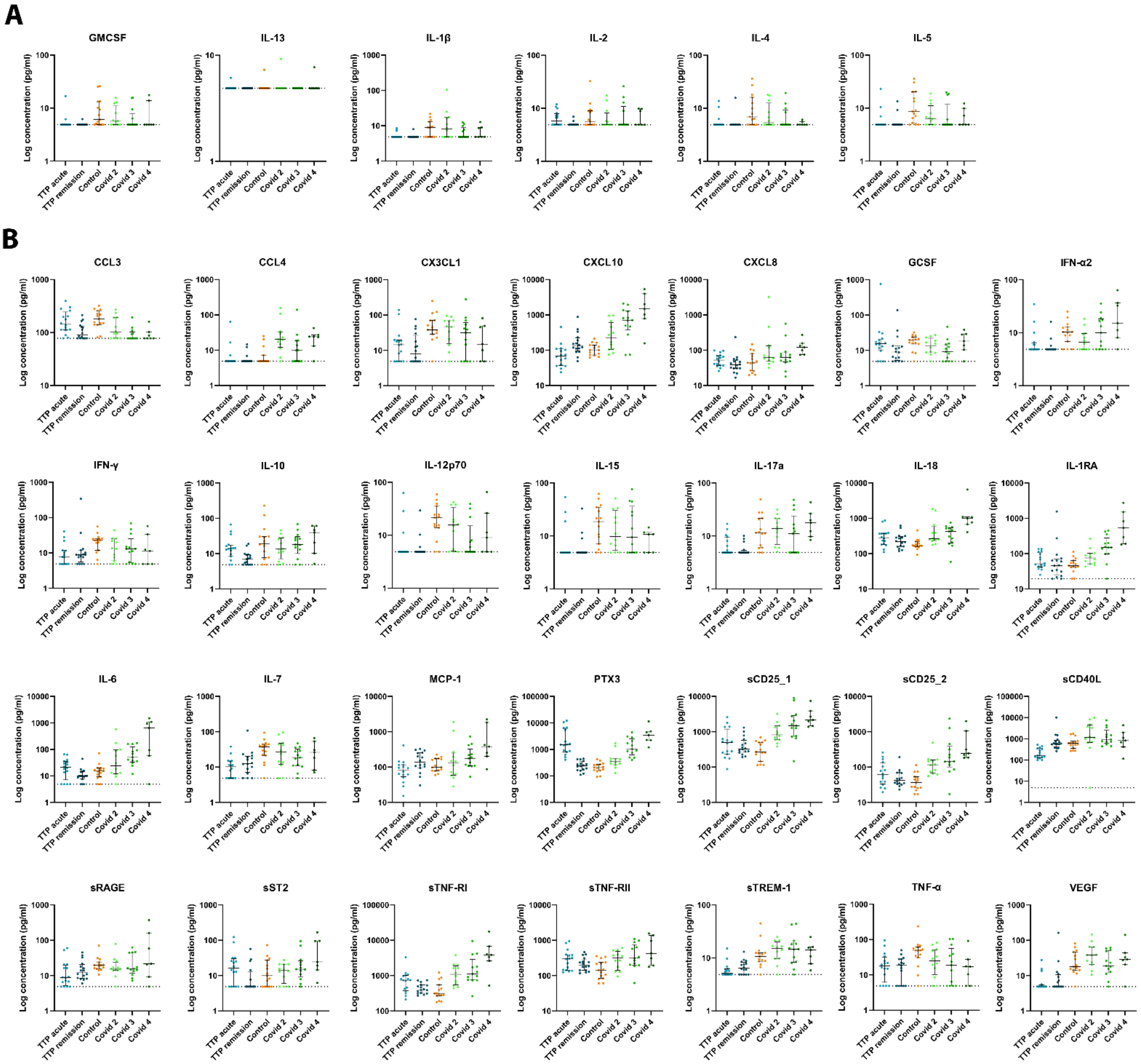

Figure S1. Concentrations of 33 SMs in TTP, Covid-19 and Control groups

The concentrations of 33 SMs were measured in six patient groups using the multiplex Legendplex array. The figure shows the logarithmic (Log) concentration values of SMs in pg/ml. sCD25 was measured twice with two different Legendplex kits, referred to as sCD25_1 and sCD25_2 in **Panel B**. SMs below the detection limit (indicated by the dotted line) are plotted in **Panel A**, while those above the detection limit are shown in **Panel B**.

Figure S2.

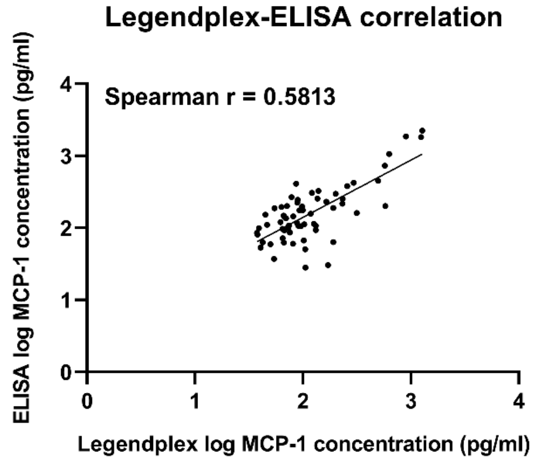

Figure S2. Correlation of MCP-1 concentrations measured by Legendplex and ELISA

The logarithmically transformed concentration values of MCP-1, measured by Legendplex and ELISA, were plotted. Spearman correlation was applied, yielding r = 0.5813, p < 0.0001.

Figure S3.

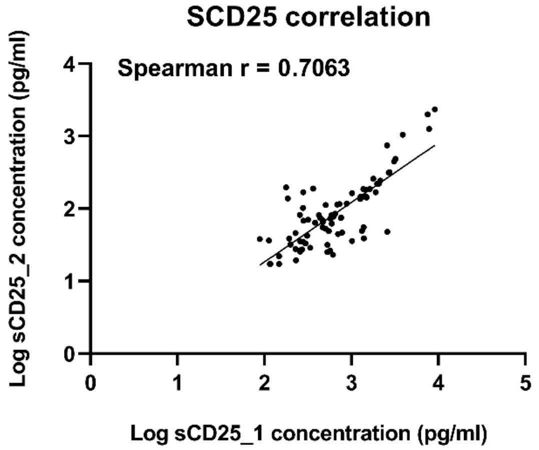

Figure S3. Correlation of sCD25 concentrations measured by the Human Inflammation Panel and the Human Covid-19 Cytokine Storm Panel 2 Legendplex kits

The logarithmically transformed concentration values of sCD25, measured by two different Legendplex kits (referred to as sCD25_1 and sCD25_2) were plotted. Spearman correlation was applied, yielding r = 0.7063, p < 0.0001.

Figure S4.

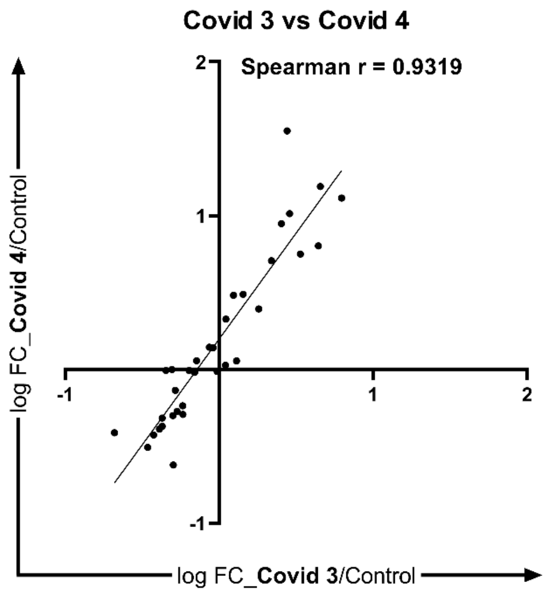

Figure S4. Correlation of SM concentrations of Covid 3 and Covid 4 groups

The logarithmically transformed fold change (FC) values (Covid 3-to-control ratio and Covid 4-to-control ratio) of the 33 SMs were plotted. Spearman correlation was applied, yielding r = 0.9319, p < 0.0001.

SUPPLEMENTARY TABLES

Table S1. Spearman correlation of age and SM concentrations. The p-value threshold of 0.000255 was determined after applying a 5% false discovery rate correction using the Benjamini-Hochberg method. na = not applicable

Soluble mediator	Spearman r/ p	TTP acute	TTP remission	Control	Covid 2	Covid 3	Covid 4
**CCL3**	r	-0.3191	0.2296	-0.6972	-0.08834	0.2638	-0.6301
	p	0.227	0.3895	0.0049	0.785	0.3787	0.1429
**CCL4**	r	0.2232	0.01733	-0.4682	0.04211	0.1449	0.7143
	p	0.4128	0.9524	0.0802	0.8992	0.632	0.0881
**CX3CL1**	r	-0.02099	-0.4965	-0.6236	-0.5018	0.1257	-0.9009
	p	0.9398	0.0526	0.0148	0.0988	0.6783	0.0095
**CXCL10**	r	-0.02511	-0.07829	-0.08266	-0.007018	-0.2176	-0.1071
	p	0.9276	0.7724	0.769	0.9869	0.4719	0.8397
**CXCL8**	r	0.1344	-0.03841	-0.611	0.3333	0.5096	0.8214
	p	0.6174	0.8884	0.0175	0.2875	0.0776	0.0341
**GCSF**	r	-0.1244	-0.03869	-0.487	0.3789	0.3143	-0.3214
	p	0.6438	0.8863	0.0675	0.2236	0.2917	0.4976
**GMCSF**	r	-0.005136	-0.2532	-0.205	-0.3711	0.2389	0.2673
	p	0.9999	0.5	0.4599	0.233	0.4306	0.5714
**IFN-α2**	r	-0.2546	-0.2232	-0.642	-0.1892	0.4064	-0.07143
	p	0.3379	0.4086	0.0116	0.5516	0.167	0.9063
**IFN-γ**	r	-0.2128	-0.05952	-0.823	-0.08787	0.2105	-0.8154
	p	0.4247	0.8252	0.0003	0.784	0.4863	0.0381
**IL-10**	r	-0.08795	0.1829	-0.5755	0.1018	0.2303	-0.4643
	p	0.7437	0.4941	0.0268	0.7527	0.4442	0.3024
**IL-12p70**	r	0.1018	0.2665	-0.3786	-0.3428	0.2096	0.03706
	p	0.7107	0.3226	0.1633	0.2731	0.4872	0.969
**IL-13**	r	-0.2532	na	-0.4357	0.1315	na	0.2041
	p	0.5625	na	0.1333	0.8333	na	0.8571
**IL-15**	r	-0.01083	-0.247	-0.4344	-0.2792	0.3032	-0.2594
	p	0.9753	0.3649	0.1063	0.3761	0.3104	0.5905
**IL-17A**	r	-0.1074	-0.1184	-0.598	-0.007018	0.4912	-0.07143
	p	0.69	0.6609	0.0205	0.9869	0.09	0.9063
**IL-18**	r	0.04136	-0.2555	0.07368	0.3193	0.1846	0.5
	p	0.8797	0.3369	0.7938	0.3092	0.5432	0.2667
**IL-1RA**	r	0.2393	-0.1183	-0.4568	0.1754	0.1267	-0.2143
	p	0.3693	0.6599	0.088	0.5832	0.6785	0.6615
**IL-1β**	r	-0.1322	-0.2532	-0.4982	-0.1055	0.1413	0.5714
	p	0.6339	0.5	0.061	0.7431	0.6411	0.2
**IL-2**	r	-0.05364	0.3325	-0.3902	0.1604	0.3533	0.07881
	p	0.8422	0.2292	0.1502	0.6167	0.236	0.8667
**IL-4**	r	-0.1712	-0.2532	-0.5472	-0.3558	0.1581	0.2673
	p	0.5298	0.5	0.0373	0.2523	0.6056	0.5714
**IL-5**	r	0.1127	-0.07801	-0.2994	-0.2286	0.07066	0.09851
	p	0.6827	0.7783	0.2761	0.4688	0.8206	0.819
**IL-6**	r	-0.06994	-0.2148	-0.241	0.3263	0.1653	-0.2857
	p	0.7951	0.4212	0.383	0.2983	0.587	0.556
**IL-7**	r	-0.1236	-0.1037	-0.6092	0.03691	0.2893	-0.4286
	p	0.6445	0.7006	0.0179	0.9097	0.335	0.3536
**MCP-1**	r	0.3043	0.1521	-0.04313	0.3368	-0.5399	-0.3929
	p	0.2501	0.5712	0.8795	0.2825	0.0596	0.3956
**PTX3**	r	-0.3102	0.2083	0.1509	0.7053	-0.135	0.03571
	p	0.2407	0.436	0.5889	0.0128	0.6583	0.9635
**sCD25**	r	0.1167	-0.1743	0.2264	0.6175	0.3912	-0.1786
	p	0.665	0.5158	0.4142	0.0359	0.1859	0.7131
**sCD40L**	r	0.1566	-0.03545	0.1545	-0.2246	0.2314	0.1429
	p	0.5599	0.8971	0.5799	0.48	0.4437	0.7825
**sRAGE**	r	0.008185	-0.6942	-0.6236	-0.3158	-0.1983	-0.3214
	p	0.9768	0.0037	0.0148	0.315	0.513	0.4976
**sST2**	r	0.06747	-0.3502	-0.5618	-0.106	0.01928	0.1429
	p	0.8032	0.1834	0.0318	0.742	0.9528	0.7825
**sTNF-R I**	r	0.2939	-0.1728	0.1959	0.2842	0.7107	0.5714
	p	0.2671	0.5193	0.4813	0.3677	0.0082	0.2
**sTNF-R II**	r	0.3737	0.2467	-0.1617	0.2421	0.5124	-0.03571
	p	0.1537	0.3543	0.5621	0.4452	0.0758	0.9635
**sTREM-1**	r	0.6974	0.4993	-0.3666	-0.1754	0.3251	-0.7857
	p	0.0038	0.051	0.1785	0.5832	0.2765	0.048
**TNF-α**	r	-0.1153	0.1304	-0.7044	-0.003515	0.4735	-0.5766
	p	0.6674	0.6274	0.0044	0.9934	0.1032	0.1865
**VEGF**	r	-0.03881	-0.3483	-0.4654	-0.1895	0.3609	0.4643
	p	0.8867	0.1851	0.082	0.5528	0.2245	0.3024

Table S2. Spearman correlation of sampling time and SM concentrations in Covid severity groups. P values lower than 0.05 are highlighted in pink. The p-value threshold of 0.00051 was determined after applying a 5% false discovery rate correction using the Benjamini-Hochberg method. na = not applicable

Soluble mediator	Spearman r/ p	Covid 2	Covid 3	Covid 4
**CCL3**	r	-0.0708	0.1165	0.09436
	p	0.8279	0.7017	0.8571
**CCL4**	r	0.7768	0.6407	0.01818
	p	0.0041	0.0214	0.9889
**CX3CL1**	r	-0.08787	-0.2647	-0.4679
	p	0.7866	0.3769	0.2857
**CXCL10**	r	-0.0949	-0.08564	-0.1637
	p	0.7694	0.7806	0.7302
**CXCL8**	r	0.355	-0.0663	0.6183
	p	0.2561	0.8303	0.1476
**GCSF**	r	0.1511	-0.1275	-0.5637
	p	0.6376	0.674	0.1968
**GMCSF**	r	-0.3681	0.5837	0
	p	0.2369	0.04	0.9999
**IFN-α2**	r	-0.1312	-0.1008	-0.6001
	p	0.6823	0.74	0.1651
**IFN-γ**	r	-0.04049	-0.08889	0.07549
	p	0.9009	0.7721	0.881
**IL-10**	r	-0.06678	-0.05671	-0.1091
	p	0.8381	0.8532	0.8222
**IL-12p70**	r	-0.08142	0.4625	-0.2453
	p	0.8016	0.1128	0.6167
**IL-13**	r	-0.3951	na	-0.1039
	p	0.3333	na	0.9999
**IL-15**	r	-0.1982	0.1364	-0.151
	p	0.5336	0.6539	0.7571
**IL-17A**	r	-0.04921	0.2891	0.07274
	p	0.8813	0.3326	0.8921
**IL-18**	r	0.109	0.1602	0.2
	p	0.7358	0.5986	0.6762
**IL-1RA**	r	0.2355	-0.02762	-0.691
	p	0.4584	0.931	0.0952
**IL-1β**	r	0.09112	0.4268	0.2909
	p	0.7778	0.1459	0.5
**IL-2**	r	0.1842	-0.1957	-0.632
	p	0.5636	0.521	0.1524
**IL-4**	r	-0.1914	0.4218	0
	p	0.5454	0.1526	0.9999
**IL-5**	r	-0.2379	0.4994	0.1405
	p	0.4506	0.0858	0.781
**IL-6**	r	0.2742	-0.6519	-0.07274
	p	0.3856	0.0183	0.8921
**IL-7**	r	-0.1408	-0.08287	-0.4001
	p	0.6585	0.7878	0.3746
**MCP-1**	r	0.4148	0.08011	-0.5092
	p	0.1802	0.7948	0.2492
**PTX3**	r	-0.1195	-0.2983	-0.2546
	p	0.7107	0.3196	0.581
**sCD25**	r	-0.06327	-0.05525	-0.2
	p	0.8464	0.8589	0.6762
**sCD40L**	r	-0.05975	0.1823	-0.1455
	p	0.8554	0.5482	0.7619
**sRAGE**	r	0.1617	0.1851	-0.3637
	p	0.6136	0.5419	0.4286
**sST2**	r	-0.3894	0.3785	0.01818
	p	0.21	0.2015	0.9889
**sTNF-R I**	r	0.4042	0.06077	0.2
	p	0.1924	0.8445	0.6762
**sTNF-R II**	r	0.4745	-0.2099	0.1818
	p	0.1208	0.488	0.6984
**sTREM-1**	r	-0.07381	0.06354	-0.691
	p	0.8207	0.8373	0.0952
**TNF-α**	r	-0.1074	-0.1191	-0.3578
	p	0.7372	0.6946	0.4254
**VEGF**	r	-0.2004	-0.07459	-0.1091
	p	0.5297	0.8089	0.8222

Table S3. Comparison of SM levels between TTP patients in remission phase who received immunosuppressive therapy (corticosteroids with/without rituximab) within the last 6 months before sampling and those who had no immunosuppressive therapy during this period. The p-value threshold of 0.001515 was determined after applying a 5% false discovery rate correction using the Benjamini-Hochberg method.

Soluble mediator	TTP therapy <6 month median	TTP therapy >6 month median	Mann-Whitney p
**CCL3**	111.3635	78.1250	0.2788
**CCL4**	4.8828	4.8828	0.7128
**CX3CL1**	9.0146	9.6776	0.9999
**CXCL10**	174.1498	112.5179	0.0541
**CXCL8**	34.3030	39.7991	0.8665
**GCSF**	12.3765	4.8828	0.1075
**GMCSF**	4.8828	4.8828	0.4667
**IFN-α2**	4.8828	4.8828	0.7128
**IFN-γ**	8.4989	8.6827	0.708
**IL-10**	7.4396	5.7824	0.8493
**IL-12p70**	4.8828	4.8828	0.9999
**IL-13**	4.8828	4.8828	0.9999
**IL-15**	4.8828	4.8828	0.4462
**IL-17A**	4.8828	4.8828	0.3231
**IL-18**	241.0401	201.0294	0.3969
**IL-1RA**	4.8828	4.8828	0.9551
**IL-1β**	53.2623	45.4004	0.4667
**IL-2**	4.8828	4.8828	0.4667
**IL-4**	4.8828	4.8828	0.4667
**IL-5**	4.8828	4.8828	0.2
**IL-6**	12.0630	8.7221	0.0929
**IL-7**	18.1912	12.2078	0.23
**MCP-1**	251.5765	114.3650	0.0401
**PTX3**	255.5885	224.0729	0.9551
**sCD25**	322.4804	336.5019	0.8665
**sCD40L**	578.9656	903.9460	0.1206
**sRAGE**	10.9761	13.9613	0.7789
**sST2**	4.8828	4.8828	0.6998
**sTNF-R I**	427.2336	414.0779	0.6943
**sTNF-R II**	279.8770	141.5412	0.014
**sTREM-1**	6.1359	7.4065	0.625
**TNF-α**	20.2764	13.1613	0.625
**VEGF**	4.8828	11.1009	0.1667

Table S4. SM groups in TTP (Panel A) and Covid-19 (Panel B), and comparison of the two disease groups (Panel C) based on statistical tests. Cells highlighted in green indicate an increase, those in red represent a decrease, and yellow signifies no change based on appropriate statistical tests. P value thresholds (as indicated in the table) were determined after applying a 5% false discovery rate correction using the Benjamini-Hochberg method. Values below the thresholds are highlighted in pink. na = not applicable

A

TTP	Soluble mediator	TTP remission median/ Control median	TTP acute median / TTP remission median	TTP remission vs Control p value (Mann-Whitney test) p treshold = 0.0263	TTP acute vs TTP remission p value (Wilcoxon test) p treshold = 0.0107
TTP acute ↑ than Control and TTP remission	PTX3	1.1667	6.3273	0.4008	0.0001
	sTNF-R I	1.2744	1.8571	0.2316	0.0092
No change in TTP	sST2	0.4900	3.3450	0.0863	0.1748
	sCD25	1.2094	1.5251	0.2814	0.3755
	sTNF-R II	1.3926	1.4905	0.0933	0.1297
	IL-18	1.2956	1.2976	0.2020	0.0654
	IL-1RA	1.0068	1.1162	0.9772	0.1439
	sRAGE	0.6385	0.7100	0.0366	0.1754
TTP acute ↓ than Control and TTP remission	sCD40L	0.9515	0.2805	0.7112	0.0001
Both TTP remission and TTP acute ↓ than Control, but no difference between them	sTREM-1	0.6019	0.8046	0.0009	0.2661
	IFN-α2	0.4740	–	0.0004	na
	IL-17A	0.4229	–	0.0001	na
	IFN-γ	0.3883	0.8676	0.0081	0.8501
	TNF-α	0.3857	0.9336	0.0015	0.3258
	VEGF	0.2730	–	0.0004	na
	IL-15	0.2619	–	0.0002	na
	IL-12p70	0.2290	–	0.0001	na
	CX3CL1	0.2064	1.8298	0.0012	0.5830
	IL-7	0.3398	0.8028	0.0032	0.0494

B

COVID-19	Soluble mediator	Covid median/ Control median	Covid 3-4 median/ Control median	Covid vs Control p value (Mann-Whitney test) p treshold = 0.0222	Covid trend p value (Jonckheere test) p treshold = 0.0464
Covid ↑ than Control and severity trend	CXCL10	6.7189	8.0131	0.0001	4.7885E-07
	sCD25	5.2394	6.6671	0.0001	2.5534E-08
	PTX3	4.6596	9.1333	0.0001	1.2812E-10
	sTNF-R I	4.5916	5.6104	0.0001	7.8498E-07
	IL-6	3.7695	4.9636	0.0006	0.0000
	CCL4	3.7042	2.7511	0.0014	0.0195
	IL-1RA	3.1615	4.2158	0.0001	2.8370E-08
	IL-18	2.4717	3.0644	0.0001	1.2755E-06
	sTNF-R II	2.2643	2.2643	0.0007	0.0004
	CXCL8	1.6482	1.7099	0.0206	0.0072
Covid ↑ than Control, but no severity trend	sCD40L	1.6968	1.4340	0.0139	0.1287
No change in Covid	MCP-1	1.8355	2.1026	0.0503	na
	sST2	1.5360	1.8037	0.198	na
	VEGF	1.5023	1.3822	0.3475	na
	sTREM-1	1.3564	1.3564	0.1487	na
	IL-17A	1.0991	1.0564	0.8609	na
	IL-10	0.9838	1.1646	0.7951	na
	CX3CL1	0.9520	0.6741	0.2348	na
	IFN-α2	0.8544	1.1857	0.7755	na
	sRAGE	0.8232	0.8645	0.4594	na
	GCSF	0.6356	0.6115	0.1026	na
	IL-7	0.6072	0.5414	0.1781	na
	IFN-γ	0.5611	0.5256	0.0972	na
	IL-15	0.5434	0.5434	0.1646	na
	TNF-α	0.3862	0.3501	0.0351	na
	IL-12p70	0.3646	0.2556	0.0369	na
Covid ↓ than Control and severity trend	CCL3	0.4621	0.4327	0.0001	0.0000

C

Covid 3-4 vs TTP	Soluble mediator	Covid 3-4 median / Control median	TTP acute median / Control median	Covid 3-4 median / TTP acute median	Covid 3-4 vs TTP acute p value (Mann-Whitney test) p treshold = 0.0315
Covid 3-4 ↑ than TPP acute	CXCL10	8.0131	0.6788	11.8031	0.0001
	sCD40L	1.4340	0.2669	5.3716	0.0001
	VEGF	1.3822	0.2730	5.0628	0.0001
	IL-1RA	4.2158	1.1238	3.7512	0.0001
	sCD25	6.6671	1.8446	3.6142	0.0002
	IL-6	4.9636	1.4037	3.5359	0.0003
	sTREM-1	1.3564	0.4843	2.8004	0.0001
	CCL4	2.7511	1.0000	2.7511	0.0061
	MCP-1	2.1026	0.8361	2.5147	0.0002
	IFN-α2	1.1857	0.4740	2.5014	0.0076
	IL-17A	1.0564	0.4229	2.4975	0.0127
	sTNF-R I	5.6104	2.3668	2.3704	0.0019
	IL-7	0.5414	0.2728	1.9842	0.0073
	sRAGE	0.8645	0.4534	1.9067	0.0233
	IL-18	3.0644	1.6813	1.8226	0.0215
	CXCL8	1.7099	1.2030	1.4212	0.0256
TTP acute ↑ than Covid 3-4	CCL3	0.4327	0.7925	0.5460	0.0001
No difference between Covid 3-4 and TTP acute	IL-12p70	0.2556	0.2290	1.1161	0.1000
	TNF-α	0.3501	0.3601	0.9724	0.981
	CX3CL1	0.6741	0.3778	1.7842	0.2828
	IFN-γ	0.5256	0.3369	1.5603	0.1576
	IL-15	0.5434	0.2619	2.0744	0.0397
	IL-10	1.1646	0.7263	1.6033	0.1137
	sST2	1.8037	1.6392	1.1003	0.425
	GCSF	0.6115	0.7993	0.7649	0.3371
	PTX3	9.1333	7.3826	1.2371	0.9374
	sTNF-R II	2.2643	2.0758	1.0908	0.1317

Table S5. Importance of SMs in each of the three principal components from principal component analysis of the Control and TTP remission (Panel A), TTP acute and TTP remission (Panel B), Control and Covid 3-4 (Panel C), and Covid 3-4 and TTP acute (Panel D) groups. Values are listed in decreasing order based on PC1 (**Panel A, C and D**) or PC2 (**Panel B**), the corresponding PC that differentiated between the two studied groups the best. The 3 SMs with the highest absolute values are highlighted.

A

Control vs TTP remission			
SM	PC1	PC2	PC3
**IL-15**	**0.3558**	-0.055	0.0216
**IFN-α2**	**0.3517**	-0.100	-0.050
**IL-12p70**	**0.3466**	-0.135	0.0546
IL-17A	0.3300	-0.051	-0.044
TNF-α	0.3233	0.0025	-0.255
IL-7	0.3176	0.1297	-0.128
VEGF	0.3151	0.1214	0.1156
sTREM-1	0.3047	-0.270	0.0398
IFN-γ	0.2229	0.1581	-0.382
CX3CL1	0.2153	-0.262	0.5387
PTX3	-0.087	-0.398	-0.673
sCD40L	-0.093	-0.582	0.0369
sTNF-RI	-0.102	-0.518	0.0573

B

TTP acute vs TTP remission			
SM	PC1	PC2	PC3
**PTX3**	0.0003	**0.5006**	-0.409
**CX3CL1**	0.1186	**0.4912**	0.4185
**sTNF-RI**	0.1791	**0.3879**	-0.072
sRAGE	0.2312	0.3067	0.5203
IL-17A	-0.207	0.2675	0.0973
IFN-α2	-0.415	0.1504	0.0393
IL-15	-0.413	0.1383	0.0700
IL-12p70	-0.367	0.1272	0.1312
TNF-α	-0.337	-0.002	0.1232
IFN-γ	-0.309	-0.136	0.1334
VEGF	-0.324	-0.162	0.2549
sTREM-1	0.1158	-0.204	0.1244
sCD40L	0.2235	-0.217	0.4843

C

Control vs Covid 3-4			
SM	PC1	PC2	PC3
**sTNF-RI**	**0.3489**	0.0830	0.0803
**sCD25**	**0.3395**	0.1297	0.0109
**IL-1RA**	**0.3368**	-0.141	-0.102
IL-18	0.3252	-0.162	0.0905
CXCL10	0.3198	0.0273	-0.121
PTX3	0.3138	0.1669	-0.251
sTNF-RII	0.3029	0.0390	-0.167
IL-6	0.2991	-0.223	-0.362
CCL4	0.2436	-0.227	0.4373
CXCL8	0.2125	-0.483	0.3382
sCD40L	0.1203	0.3780	0.6560
CCL3	-0.206	-0.649	0.0338

D

Covid 3-4 vs TTP acute			
SM	PC1	PC2	PC3
CCL3	0.1316	0.5666	0.0463
sRAGE	-0.196	-0.226	0.1428
sCD40L	-0.213	-0.255	-0.338
IL-17A	-0.215	0.2677	0.2291
IL-7	-0.216	0.3290	-0.384
CXCL8	-0.217	0.2984	-0.048
CCL4	-0.227	0.2357	0.2479
IFN-α2	-0.245	0.2046	-0.344
sCD25	-0.245	-0.136	0.2121
sTREM-1	-0.250	-0.209	-0.357
IL-18	-0.253	0.1695	0.3339
MCP-1	-0.254	-0.178	0.0317
sTNF-RI	-0.255	-0.142	0.3734
IL-6	-0.257	0.0302	0.0855
**VEGF**	**-0.290**	0.1766	-0.199
**IL-1RA**	**-0.295**	-0.070	0.0760
**CXCL10**	**-0.301**	-0.124	-0.047
